# c-Fos importance for brain development

**DOI:** 10.18632/aging.100862

**Published:** 2015-12-17

**Authors:** Fabiola N. Velazquez, Beatriz L. Caputto, François D. Boussin

**Affiliations:** Laboratoire de Radiopathologie, UMR967, Université Paris VII, Université Paris XI, INSERM, CEA, Fontenay-aux-Roses, France; CIQUIBIC (CONICET), Departamento de Química Biológica, Facultad de Ciencias Químicas, Universidad Nacional de Córdoba, Córdoba, Argentina

**Keywords:** c-Fos, AP-1, neurogenesis, neural progenitors

c-Fos is an immediate early response gene involved in cell proliferation and differentiation after extracellular stimuli, whereas its deregulation has been associated to oncogenic progression. As other members of the FOS family proteins (Fra-1, Fra-2, FosB) and proteins of the ATF family, c-Fos can heterodimerize with members of the JUN family (c-Jun, JunB, JunD) to form the transcription factor activator protein 1 (AP-1). As an AP-1 heterodimer, c-Fos is considered a master switch that transduces short-term stimuli into long-term responses. Independently of its AP-1 activity, c-Fos is also an activator of phospholipid synthesis at the cytoplasmic level in events demanding high rates of membrane biogenesis, as occurs for example in the exacerbated growth of tumor cells [1].

Only ∼40% of *c-fos* −/− mouse embryos survive until birth showing the importance of c-Fos for development. Moreover, surviving animals live to an average age of 6–7 months, show growth retardation, severe osteopetrosis, delayed or absent gametogenesis and altered haematopoiesis. Decrease of *c-fos* expression leading to modifications in the composition of AP-1 factors (particularly increasing the Jun/Jun homodimers) has been associated with cellular replicative senescence [2], suggesting a possible involvement of cell senescence in premature death of *c-fos* −/− mice.

c-Fos is currently used as a marker of neuronal activity and has been associated with a number of neural and behavioral responses to acute stimuli expression. In fact, age-related changes in neuronal function and plasticity with a concomitant decrease in the level of c-Fos have been observed in different brain regions [3]. In addition to the above-mentioned alterations, the *c-fos* −/− mice show behavioral alterations such as learning impairment, hyperactivity and abnormal sexual behavior. In spite of all these evidences that show a relationship between c-Fos expression and neuronal function, its role in brain development has been poorly investigated. We have recently reported that c-Fos plays an important and unsuspected role in neurogenesis [4].

Adult *c-fos* −/− mice present a ∼40–60% reduction in their body weight and their brain size is significantly smaller as compared to *c-fos +/+* mice. We have shown that this correlates with a decreased brain cell number. We have thus examined the importance of c-Fos for neo-cortex development by comparing these two types of mice at different embryonic stages. *c-fos* −/− embryos present a ∼15% reduction in the thickness of the developing cortex as compared to *wt* controls. Immuno-staining revealed that this was related to a decrease in neurogenesis in *c-fos* −/− mice during embryonic development. Indeed we showed both a small but significant increase in the number of neural progenitors and a major decrease in neuronal cells in the developing cortex of *c-fos* −/− embryos as compared to *c-fos +/+* ones. Together with an increased apoptosis, this suggests that the absence of c-Fos alters the production of neurons in knockout animals. Consistently, we showed that neural progenitors isolated from cerebral cortex of *c-fos* −/− embryos were less capable to differentiate *in vitro* into neurons than *wt* neural progenitors, highlighting the importance of c-Fos for neurogenesis in the developing brain.

c-Fos is normally expressed in a large number of cells in the developing cortex. To determine whether the importance of c-Fos for cortical development was linked to its activity as a lipid synthesis activator or as an AP-1 transcription factor, both activities were measured in the developing cortex. No difference in phospholipid synthesis was observed between cortical extracts from *c-fos* −/− and *c-fos +/+* embryos even after addition of recombinant c-Fos to the assay samples. Therefore this indicates that the membrane biosynthesis machinery produces membrane at a rate high enough to be independent of its activation by c-Fos in the developing cortex.

Interestingly, we showed that the absence of *c-fos* increases the content of AP-1/DNA complexes in cortical extracts, without affecting c-Jun expression. It is still unknown whether different combinations of AP-1 components regulate specific neural genes differently or whether a single combination of AP-1 plays a more general role in various neural genes [5]. Our results suggests that the lack of *c-fos* during cerebral cortex development provokes a modification in the content and/or stability of the AP-1 transcription factor dimers that cannot efficiently replace the loss of c-Fos and leads to abnormal cortical development of *c-fos* −/− mice.

Altogether, our data highlight the importance of c-Fos-containing AP-1 transcription factors for efficient neurogenesis during cortical development. Further experiments should thus investigate the transcription regulatory activity of c-Fos containing and non-containing AP-1 factors in neural progenitors and their specific importance at different developmental stages or for specific neuronal lineages.
